# Benefits of upgrading right ventricular to biventricular pacing in heart failure patients with atrial fibrillation

**DOI:** 10.1093/europace/euae179

**Published:** 2024-07-09

**Authors:** Béla Merkely, Robert Hatala, Eperke Merkel, Mátyás Szigeti, Boglárka Veres, Alexandra Fábián, István Osztheimer, László Gellér, Michal Sasov, Jerzy K Wranicz, Csaba Földesi, Gábor Duray, Scott D Solomon, Valentina Kutyifa, Attila Kovács, Annamária Kosztin

**Affiliations:** Heart and Vascular Center, Semmelweis University, Varosmajor 68, H-1122 Budapest, Hungary; Department of Cardiology and Angiology, National Institute of Cardiovascular Diseases, Slovak Medical University, Bratislava, Slovakia; Heart and Vascular Center, Semmelweis University, Varosmajor 68, H-1122 Budapest, Hungary; Heart and Vascular Center, Semmelweis University, Varosmajor 68, H-1122 Budapest, Hungary; Heart and Vascular Center, Semmelweis University, Varosmajor 68, H-1122 Budapest, Hungary; Heart and Vascular Center, Semmelweis University, Varosmajor 68, H-1122 Budapest, Hungary; Heart and Vascular Center, Semmelweis University, Varosmajor 68, H-1122 Budapest, Hungary; Heart and Vascular Center, Semmelweis University, Varosmajor 68, H-1122 Budapest, Hungary; Department of Cardiology and Angiology, National Institute of Cardiovascular Diseases, Slovak Medical University, Bratislava, Slovakia; Department of Electrocardiology, Medical University of Lodz, Lodz, Poland; Department of Cardiology, Gottsegen National Cardiovascular Center, Budapest, Hungary; Department of Cardiology, Central Hospital of Northern Pest—Military Hospital, Budapest, Hungary; Cardiovascular Division, Brigham and Women's Hospital, Harvard Medical School, Boston, MA, USA; Heart and Vascular Center, Semmelweis University, Varosmajor 68, H-1122 Budapest, Hungary; Clinical Cardiovascular Research Center, University of Rochester, Rochester, NY, USA; Heart and Vascular Center, Semmelweis University, Varosmajor 68, H-1122 Budapest, Hungary; Heart and Vascular Center, Semmelweis University, Varosmajor 68, H-1122 Budapest, Hungary

**Keywords:** Cardiac resynchronization therapy, Upgrade, Right ventricular pacing, Pacing-induced cardiomyopathy, Heart failure, Atrial fibrillation

## Abstract

**Aims:**

Recommendations on cardiac resynchronization therapy (CRT) in patients with atrial fibrillation or flutter (AF) are based on less robust evidence than those in sinus rhythm (SR). We aimed to assess the efficacy of CRT upgrade in the BUDAPEST-CRT Upgrade trial population by their baseline rhythm.

**Methods and results:**

Heart failure patients with reduced ejection fraction (HFrEF) and previously implanted pacemaker (PM) or implantable cardioverter defibrillator (ICD) and ≥20% right ventricular (RV) pacing burden were randomized to CRT with defibrillator (CRT-D) upgrade (*n* = 215) or ICD (*n* = 145). Primary [HF hospitalization (HFH), all-cause mortality, or <15% reduction of left ventricular end-systolic volume] and secondary outcomes were investigated. At enrolment, 131 (36%) patients had AF, who had an increased risk for HFH as compared with those with SR [adjusted hazard ratio (aHR) 2.99; 95% confidence interval (CI) 1.26–7.13; *P* = 0.013]. The effect of CRT-D upgrade was similar in patients with AF as in those with SR [AF adjusted odds ratio (aOR) 0.06; 95% CI 0.02–0.17; *P* < 0.001; SR aOR 0.13; 95% CI 0.07–0.27; *P* < 0.001; interaction *P* = 0.29] during the mean follow-up time of 12.4 months. Also, it decreased the risk of HFH or all-cause mortality (aHR 0.33; 95% CI 0.16–0.70; *P* = 0.003; interaction *P* = 0.17) and improved the echocardiographic response (left ventricular end-diastolic volume difference −49.21 mL; 95% CI −69.10 to −29.32; *P* < 0.001; interaction *P* = 0.21).

**Conclusion:**

In HFrEF patients with AF and PM/ICD with high RV pacing burden, CRT-D upgrade decreased the risk of HFH and improved reverse remodelling when compared with ICD, similar to that seen in patients in SR.

## Graphical Abstract

**Graphical Abstract:**
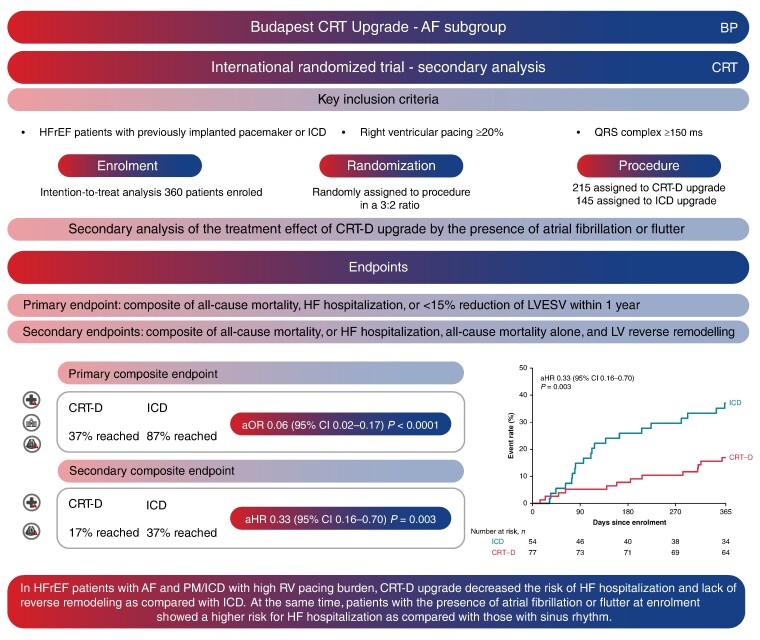
In AF patients CRT-D upgrade reduced the risk of mortality or HF hospitalization as compared with ICD, additionally echocardiographic parameters, symptoms, and NT-proBNP levels improved. However, the risk of HF hospitalization was higher in AF patients when compared with SR. AF, atrial fibrillation or flutter; aOR, adjusted odds ratio; aHR, adjusted hazard ratio; CI, confidence interval; CRT-D, cardiac resynchronization therapy with defibrillator; ICD, implantable cardioverter defibrillator; HF, heart failure; HFrEF, heart failure with reduced ejection fraction; LVESV, left ventricular end-systolic volume; NT-proBNP, N-terminal pro b-type natriuretic peptide; PCI, percutaneous coronary intervention; PM, pacemaker; RV, right ventricular; SR, sinus rhythm.

**What’s new?**

- Patients in the BUDAPEST-CRT Upgrade trial showed a substantial treatment effect of cardiac resynchronization therapy with defibrillator (CRT-D) on the primary outcome regardless of the baseline rhythm.
- In the subgroup of atrial fibrillation or flutter (AF) patients: a clear benefit of CRT-D upgrade compared with implantable cardioverter defibrillator.
- Patients with AF are at a higher risk of heart failure (HF) hospitalization as compared with patients with sinus rhythm.
- Atrial fibrillation or flutter patients in the CRT-D arm experienced improvements in echocardiographic parameters, N-terminal pro b-type natriuretic peptide levels, and HF symptoms.

## Introduction

Atrial fibrillation and flutter (AF) are the most common sustained arrhythmias of the adult population worldwide, frequently affecting patients with heart failure (HF), in whom the prevalence is between 30 and 45%.^1^

Additionally, in patients with a previously implanted pacemaker (PM) or implantable cardioverter defibrillator (ICD), AF burden is still higher as compared with those without a device; moreover, the right ventricular (RV) pacing burden can further increase the risk of AF.^2–4^

Despite AF being a frequent condition in HF, data on the efficacy of cardiac resynchronization therapy (CRT) in patients with AF are relatively scarce. Since there has been no large, randomized, controlled trial directly designed to investigate the difference in the treatment effect of CRT in sinus rhythm (SR) vs. AF, the current guidelines provide a IIa level of evidence C for patients with left ventricular ejection fraction (LVEF) < 35%, QRS > 130 ms, and New York Heart Association (NYHA) III–IV without any other specific details for QRS morphology, width or mild symptoms as can be seen for those with SR.^5^ These recommendations are based on the subgroup analyses of those landmark *de novo* CRT trials that investigated mainly patients in SR or those with AF in their medical history or those trials that investigated permanent AF patients but with uncertain benefit.^6–10^

The ‘**B**iventricular **U**pgrade on left ventricular reverse remodelling and clinical outcomes in patients with left ventricular **D**ysfunction and intermittent or permanent **AP**ical/**Se**p**T**al right ventricular pacing (BUDAPEST) Upgrade CRT’ trial was the first, which investigated the efficacy of CRT upgrade in patients with intermittent or permanent right ventricular (RV) pacing and LV reduced ejection fraction (HFrEF). Despite the advanced-stage HF cohort, the trial provided robust data favouring CRT with defibrillator (CRT-D) as compared with ICD alone in the combined risk of all-cause mortality, heart failure hospitalization, or absence of reverse remodeling.^11^ In this patient population, the incidence of AF was outstandingly high, and almost two-thirds of the patients had current or history of AF with a high proportion of RV pacing.

Therefore, in the current analysis, we provide data on the efficacy of CRT upgrade during the 12-month follow-up in AF patients as compared with SR on the basis of baseline rhythm.

## Methods

### Study population

The BUDAPEST-CRT Upgrade trial was an investigator-initiated prospective, multicentre, randomized, controlled trial. The design, protocol, and the primary results of the BUDAPEST-CRT Upgrade trial have been previously published.^11–13^ In total, 360 patients were enrolled and randomly assigned to CRT-D upgrade (*n* = 215) or ICD (*n* = 145) in a 3:2 ratio, in 17 sites from Europe and Israel. Patients had been already implanted with a PM or ICD for at least 6 months prior to enrolment, reduced LVEF (≤35%), HF symptoms (NYHA functional class II–IVa), wide-paced QRS (>150 ms), and ≥20% of RV pacing burden and treated with guideline-directed medical therapy without having a native intrinsic left bundle branch block. The hereby studied patient population was defined by whether the patients presented with atrial fibrillation or flutter at enrolment as it has been previously specified in the statistical analysis plan. However, additional analyses were also performed by the presence and history of atrial fibrillation or flutter (see Supplementary material). The study protocol was approved by local and institutional ethics committees.

### Data and follow-up

Enrolled patients were followed up for 12 months after randomization. Outpatient follow-up visits were done at 1, 6, and 12 months when clinical parameters, electrocardiogram, device interrogation, echocardiographic, and biochemical parameters were collected. Additionally, 6-min walk test (6-MWT) and EQ-5D-3L quality of life questionnaires were also mandatory at baseline and the 12-month follow-up.

Echocardiographic data were submitted to the Echocardiography Core Laboratory for central assessment (Semmelweis University, Heart and Vascular Center, Budapest, Hungary). Left ventricular volumes and ejection fraction were calculated using the biplane Simpson method, baseline visit (after randomization and before implantation), and at the 12-month follow-up visit.

### Endpoints

The primary composite endpoint was defined as the first occurrence of HF hospitalization, all-cause mortality within 1 year, or less than 15% reduction in LV end-systolic volume (LVESV) at 12 months assessed by echocardiography. Secondary endpoints were the composite of all-cause mortality or HF hospitalizations, all-cause mortality, and LV volume change at 12 months. The pre-specified tertiary endpoints were changes in quality of life (assessed by EQ-5D-3L questionnaire), HF symptoms (NYHA functional class), 6-MWT, and N-terminal pro b-type natriuretic peptide (NT-proBNP) from baseline to 12 months.

### Statistical analysis

Continuous variables with normal distributions are expressed as mean ± standard deviation, while those with non-normal distributions as medians with interquartile range (25th–75th percentile). Categorical variables are summarized with frequencies and percentages. Baseline clinical characteristics were compared by the baseline rhythm in both arms using a *t*-test for normally distributed continuous variables, the Mann–Whitney *U* test for non-normally distributed variables, while *χ*^2^ test was used for dichotomous variables as appropriate.

The primary outcome was analysed using logistic regression due to its binary component, and the effect size was expressed as adjusted and unadjusted odds ratios (ORs) with associated 95% confidence intervals (CIs).

Time-to-event secondary outcomes (composite endpoint of all-cause mortality and HF hospitalization and all-cause mortality alone) were analysed by Cox proportional hazard models. Heart failure hospitalization alone, as an outcome, was specified in a *post hoc* manner. Changes in LV end-diastolic volume (LVEDV), LVEF, NT-proBNP, 6-MWT, and EQ-5D-3L were analysed by linear regression. Change in NYHA class was assessed by the proportion of patients who changed class from baseline to 12 months providing a 5-scale assessment and was studied with ordinal logistic regression. When measurements of trial patients were unavailable due to death, imputed values were used as 0 m (6-MWT) or 0 score (EQ-5D-3L) or a 5th grade (NYHA class) as it was outlined in the statistical analysis plan of the main analysis.

The primary outcome analysis was done in the modified intention-to-treat (ITT) population: patients with missing results of the echocardiography component of the primary outcome and who did not meet the primary outcome through the other components (HF or death) were excluded. However, they were included in all other analyses based on ITT principles.

The pre-specified adjustment factors were age, sex, country, ischaemic aetiology, diabetes mellitus, secondary prevention ICD, and baseline NYHA class although country and baseline NYHA class were not applicable due to the small sample size in the subgroups. Estimation of the treatment effect itself in the subgroups was analysed by the application of the above-mentioned modelling strategy to each subgroup. Interaction *P*-values were also calculated to test whether the effects significantly differed between the subgroups. The presented *P*-values and the width of the CIs were not adjusted for multiplicity.

Statistical analyses were performed by using Stata version 18.0 (StataCorp., College Station, TX, USA).

## Results

### Baseline clinical characteristics by rhythm

The baseline clinical characteristics for the total patient cohort were reported previously,^13^ showing that concomitant comorbidities were found in a high proportion of patients, history or current AF in 56% (60% in the ICD arm and 54% in the CRT-D arm). At enrolment, 36% of the CRT-D arm and 37% of the ICD arm presented with atrial fibrillation, respectively (*Table 1*).

**Table 1:** Characteristics of the participants at baseline, according to randomization arm

Characteristics^a^	CRT-D (*n* = 215)		ICD (*n* = 145)	
	SR (*n* = 138)	AF (*n* = 77)	SR (*n* = 91)	AF (*n* = 54)
Age, years	72.0 ± 7.1	74.4 ± 7.6	71.2 ± 9.1	75.1 ± 6.1
Male sex, no. (%)	118 (85)	67 (87)	85 (93)	50 (93)
BMI, kg/m^2^	28.8 ± 4.8	29.5 ± 5.0	27.7 ± 4.6	28.8 ± 5.3
NYHA class, no. (%)				
II	73 (53)	32 (42)	42 (46)	22 (41)
III	59 (43)	42 (54)	47 (41)	31 (57)
IVa	6 (4)	3 (4)	2 (2)	1 (2)
6-MWT (m), mean ± SD	275 ± 110	259 ± 126	289 ± 114	279 ± 112
EQ-5D-3L score, mean ± SD	0.67 ± 0.29	0.62 ± 0.30	0.69 ± 0.28	0.67 ± 0.29
Echocardiographic parameters				
Left ventricular end-diastolic volume, mL	234 ± 80	226 ± 80	238 ± 78	204 ± 66
Left ventricular end-systolic volume, mL	176 ± 66	173 ± 67	179 ± 67	154 ± 57
Left ventricular ejection fraction, %	25.1 ± 6.9	23.9 ± 6.9	25.2 ± 6	25.5 ± 7.0
Medical history, no. (%)				
Ischaemic aetiology	83 (60)	44 (57)	53 (58)	28 (52)
AMI	68 (49)	34 (44)	43 (47)	22 (41)
CABG	37 (27)	16 (21)	21 (23)	12 (22)
PCI	54 (39)	31 (40)	35 (38)	20 (37)
Hypertension	116 (84)	62 (80)	69 (76)	42 (78)
Diabetes	53 (38)	30 (39)	28 (31)	17 (31)
Hyperlipidaemia	62 (45)	33 (43)	44 (48)	26 (48)
Asthma	5 (4)	3 (4)	2 (2)	1 (2)
Chronic obstructive pulmonary disease	18 (13)	12 (16)	13 (14)	5 (9)
Current smoking	15 (11)	3 (4)	6 (7)	1 (2)
Known valvular heart disease	22 (16)	12 (16)	17 (19)	12 (22)
Valve surgery	16 (12)	12 (15)	7 (8)	3 (5)
Cerebrovascular accident or transient ischaemic attack	15 (11)	18 (23)	11 (12)	12 (22)
Peripheral vascular disease	16 (12)	5 (6)	10 (11)	3 (5)
History of VT or VF	32 (23)	15 (19)	25 (27)	12 (22)
Heart failure hospitalization 12 months prior to enrolment	68 (49)	33 (43)	50 (55)	27 (50)
Baseline medication, no. (%)				
ACE inhibitor	104 (75)	53 (69)	70 (77)	38 (70)
ARB	22 (16)	21 (27)	15 (16)	8 (15)
ARNI				
Beta-blockers	127 (92)	70 (91)	83 (91)	48 (89)
MRA	86 (62)	48 (62)	53 (58)	38 (70)
Loop diuretics	110 (80)	60 (78)	75 (82)	43 (80)
Calcium channel blocker	19 (14)	10 (13)	7 (8)	3 (5)
Amiodarone	41 (30)	11 (14)	24 (26)	11 (20)
Digoxin	8 (6)	9 (12)	8 (9)	9 (17)
Prior device type, no. (%)				
Pacemaker	97 (70)	53 (69)	61 (67)	21 (39)
Implantable cardioverter defibrillator	40 (29)	24 (31)	29 (32)	33 (61)
Cardiac resynchronization therapy with plug	1 (1)	0 (0)	1 (1)	0 (0)
Pacemaker interrogation				
Per cent right ventricular pacing prior to enrolment, % (median/IQR)	98 (88–100)	91 (65–98)	98 (79–99)	98 (90–99)
NT-proBNP, pg/mL (median/IQR)	1793 (928–4342)	2554 (1606–3782)	2431 (1448–4549)	1923 (1275–3541)

6-MWT, 6-min walk test; ACE, angiotensin-converting enzyme; AF, atrial fibrillation or flutter; AMI, acute myocardial infarction; ARB, angiotensin receptor blocker; ARNI, angiotensin receptor–neprilysin inhibitor; BMI, body mass index; CABG, coronary artey bypass grafting; CRT-D, cardiac resynchronization therapy with defibrillator; ICD, implantable cardioverter defibrillator; MRA, mineralocorticoid receptor antagonist; NT-proBNP, N-terminal pro b-type natriuretic peptide; NYHA, New York Heart Association; PCI, percutaneous coronary intervention; SR, sinus rhythm; VF, ventricular fibrillation; VT, ventricular tachycardia.

^a^Plus–minus values are means ± SD.

Patients with atrial fibrillation were older, regardless of the treatment arm [CRT-D arm SR 72.0 ± 7.1 years vs. AF 74.4 ± 7.6 years (*P* = 0.023) and ICD arm SR 71.2 ± 9.1 years vs. AF 75.1 ± 6.1 years (*P* = 0.007)]. Comorbidity profiles were similar in both patient groups (*Table 1*). Regarding echocardiographic parameters at baseline, patients with atrial fibrillation in the ICD arm presented with a lower LVEDV and LVESV compared with patients with SR (LVEDV SR 237.6 ± 78.1 mL vs. AF 203.8 ± 65.7 mL; *P* = 0.009 and LVESV SR 179.3 ± 67.0 mL vs. AF 153.7 ± 57.3 mL; *P* = 0.031).

Patients were treated strictly with guideline-directed medical therapy,^14^ which was well balanced between patients with SR or AF. However, amiodarone was more frequently prescribed to patients in the subgroup of SR at baseline in the CRT-D arm (CRT-D arm SR 30% vs. AF 14%; *P* = 0.011).

Regarding the previously implanted devices, patients on the ICD arm with AF were more likely to have an ICD prior to enrolment than a PM (ICD arm AF PM 39% vs. ICD 61%; *P* = 0.034). Furthermore, patients with AF in the CRT-D arm had a lower percentage of RV pacing prior to enrolment compared with patients with SR [SR 98% (IQR 88–100) vs. AF 91% (IQR 65–98); *P* < 0.001].

### Treatment effect of cardiac resynchronization therapy with defibrillator upgrade by the presence of atrial fibrillation or flutter

**Figure 1:**
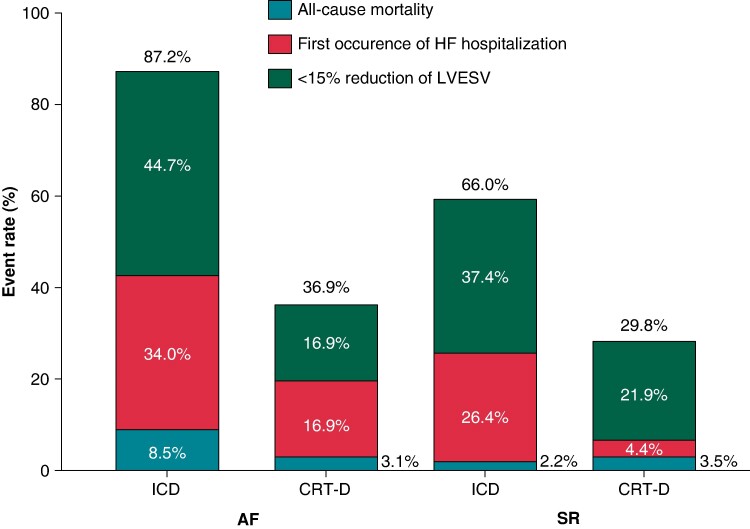
Event rate of the primary composite outcome in the ICD and CRT-D arms and its components: first occurrence of HF hospitalization with or without subsequent all-cause death, all-cause death without previous HF hospitalization, and <15% reduction in LVESV assessed at 12-month visit by echocardiography in patients without previous HF hospitalization according to the baseline rhythm. AF, atrial fibrillation or flutter; CRT-D, cardiac resynchronization therapy with defibrillator; HF, heart failure; ICD, implantable cardioverter defibrillator; LVESV, left ventricular end-systolic volume; SR, sinus rhythm.

**Figure 2:**
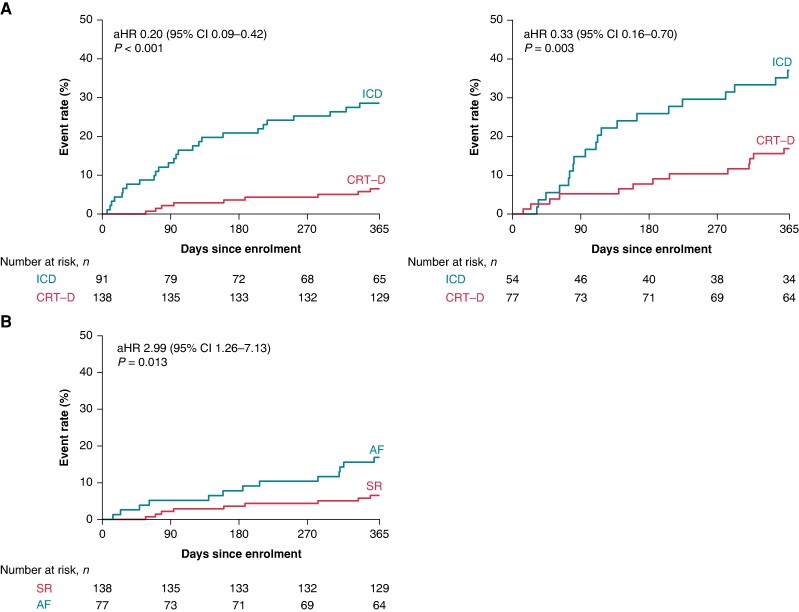
(*A*) Kaplan–Meier curves for the secondary composite outcome of first occurrence of all-cause mortality or HF hospitalization according to the baseline rhythm (SR and atrial fibrillation or flutter) in the total patient cohort. (*B*) Kaplan–Meier curves for the secondary composite outcome of first occurrence of all-cause mortality or HF hospitalization in CRT-D patients by baseline rhythm (atrial fibrillation or flutter vs. SR). AF, atrial fibrillation or flutter; aHR, adjusted hazard ratio; CI, confidence interval; CRT-D, cardiac resynchronization therapy with defibrillator; ICD, implantable cardioverter defibrillator; SR, sinus rhythm.

In the modified ITT population with AF at enrolment, the primary endpoint occurred in 65/112 (58%) patients, 41/47 (87%) in the ICD arm, and 24/65 (37%) patients in the CRT-D upgrade arm, respectively (adjusted OR 0.06; 95% CI 0.02–0.17; *P* < 0.0001; interaction *P* = 0.29) (*Figure 1*) (*Table 2*). Regarding the secondary endpoint, upgrade to CRT-D decreased the hazard of the composite of HF hospitalization and all-cause mortality (adjusted HR 0.33; 95% CI 0.16–0.70; *P* = 0.003) (*Figure 2A*), which was mainly driven by the reduction in HF hospitalization (adjusted HR 0.38; 95% CI 0.17–0.85; *P* = 0.019) (*Table 2*). Even though we observed a different treatment effect of CRT-D upgrade on HF hospitalization in the interaction model by the baseline rhythm (interaction *P* = 0.036), there was a significant benefit of CRT-D upgrade in terms of HF hospitalizations, also in patients with AF (HR 0.38, 95% CI 0.17–0.85; *P* = 0.019).

**Table 2:** Outcomes by treatment arm in patients with atrial fibrillation

Endpoints	ICD (*n* = 54)	CRT-D (*n* = 77)	Measure of effect	Unadjusted hazard or odds ratio or difference (95% CI)	*P*-value	Adjusted hazard or odds ratio or difference (95% CI)^a^	*P*-value
**Primary outcome**							
Composite endpoint of all-cause death or heart failure hospitalization or <15% end-systolic volume decrease, no./total no. (%)	41/47 (87)	24/65 (37)	OR	0.09 (0.03–0.23)	<0.0001	0.06 (0.02–0.17)	<0.0001
**Secondary outcomes**							
Composite endpoint of all-cause death or HF hospitalizations, no./total no. (%)	20/54 (37)	13/77 (17)	HR	0.39 (0.20–0.79)	0.009	0.33 (0.16–0.70)	0.003
Death from any cause, no./total no. (%)	7/54 (13)	6/77 (8)	HR	0.59 (0.20–1.76)	0.345	0.57 (0.18–1.80)	0.337
HF hospitalization^b^, no./total no. (%)	16/54 (30)	11/77 (14)	HR	0.44 (0.20–0.94)	0.034	0.38 (0.17–0.85)	0.019
Changes in left ventricular end-diastolic volume from baseline to 12 months, mL ± SD	2.08 ± 39.9	−46.9 ± 52.3	Difference	−48.95 (−68.52 to −29.38)	<0.0001	−49.21 (−69.10 to −29.32)	<0.0001
Changes in left ventricular ejection fraction from baseline to 12 months, % ± SD	−0.23 ± 7.15	9.75 ± 8.8	Difference	9.99 (6.64–13.34)	<0.0001	10.28 (6.92–13.65)	<0.0001
**Tertiary outcomes**							
Changes in 6-MWT at 12 months (m), mean ± SD	−2.93 ± 136.3	23.5 ± 123.7	Difference	26.43 (−34.41 to 87.27)	0.389	22.54 (−41.84 to 86.91)	0.487
Changes EQ-5D-3L from baseline to 12 months, score ± SD	0.005 ± 0.35	0.06 ± 0.37	Difference	0.06 (−0.08 to 0.20)	0.401	0.07 (−0.07 to 0.20)	0.322
Changes in NT-proBNP (pg/mL), from baseline to 12 months, median (25th–75th percentile)	−245 (−1073–809)	−431 (−1705–73)	Difference	−1006 (−2505 to 492)	0.185	−1645 (−3204 to −85)	0.039
Changes in NYHA class from baseline to 12 months, NYHA class			Difference	0.47 (0.23–0.99)	0.047	0.38 (0.18–0.83)	0.015
Unchanged, no./total no. (%)	21/41 (51)	33/64 (51)					
Improved, no./total no. (%)	15/41 (37)	28/64 (44)					
Worsened, no./total no. (%)	5/41 (12)	3/64 (5)					

6-MWT, 6-min walk test; CRT-D, cardiac resynchronization therapy with defibrillator; HF, heart failure; HR, hazard ratio; ICD, implantable cardioverter defibrillator; NT-proBNP, N-terminal pro b-type natriuretic peptide; NYHA, New York Heart Association; OR, odds ratio; SD, standard deviation.

^a^Adjusted for pre-specified variables considered as strong predictors of the outcome: age, sex, ischaemic aetiology, diabetes, and secondary prevention.

^b^ *Post hoc* analysis.

**Figure 3:**
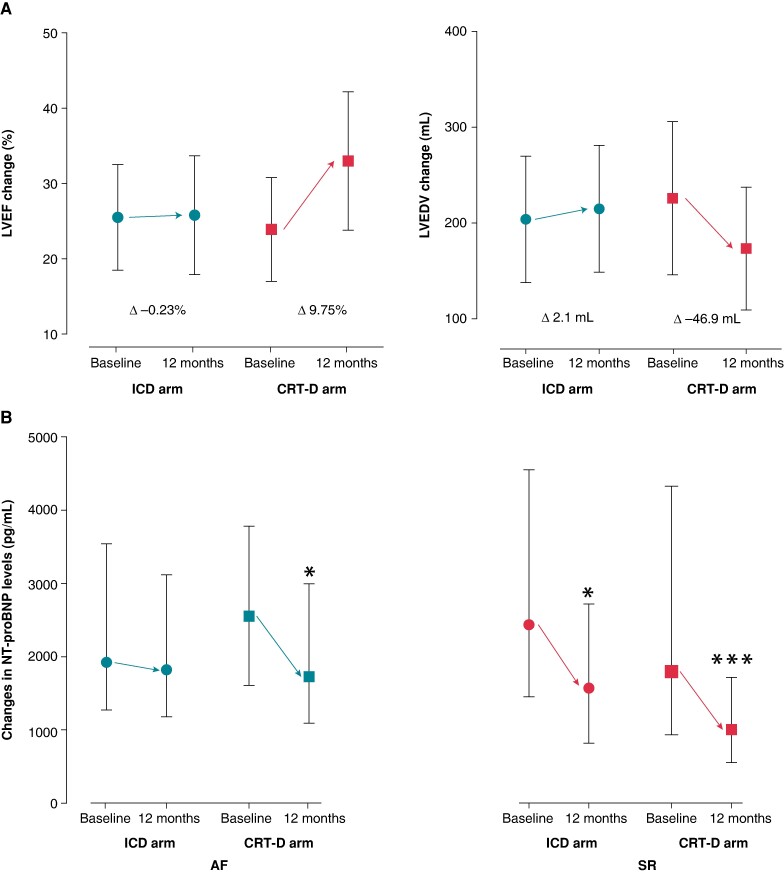
(*A*) Echocardiographic response in AF patients (LVEF and LVEDV) by treatment arms. (*B*) NT-proBNP change from baseline to 12 months according to the baseline rhythm by treatment arms. AF, atrial fibrillation or flutter; CRT-D, cardiac resynchronization therapy with defibrillator; ICD, implantable cardioverter defibrillator; LVEDV, left ventricular end-diastolic volume; LVEF, left ventricular ejection fraction; NT-proBNP, N-terminal pro b-type natriuretic peptide; SR, sinus rhythm. * *P* < 0.05; ** *P* < 0.0001.

Left ventricular morphological and functional response favoured CRT-D upgrade, LVEDV difference at 12 months decreased significantly (adjusted difference −49.21 mL; 95% CI −69.10 to −29.32; *P* < 0.0001), and LVEF ameliorated (adjusted difference 10.28%; 95% CI 6.92–13.65; *P* < 0.0001) (*Table 2*; *Figure 3A*).

Changes in NYHA functional class and natriuretic peptide levels also support the choice of CRT-D upgrade as compared with ICD alone (*Table 2*). Patients with AF undergoing upgrade procedure also show a clinical benefit by decreasing the HF symptoms [NYHA class changes (adjusted OR 0.39; 95% CI 0.18–0.83; *P* = 0.015) and by decreasing NT-proBNP levels (adjusted difference −1645 pg/mL; 95% CI −3204 to −85; *P* = 0.039)] (*Table 2*; *Figure 3B*).

However, patients did not show benefit regarding changes in quality of life (EQ-5D-3L score adjusted difference 0.07; 95% CI −0.07 to 0.20; *P* = 0.32) or exercise capacity (6-MWT changes adjusted difference 22.54 m; 95% CI −41.84 to 86.91; *P* = 0.49) (*Table 2*).

The beneficial effect of CRT-D upgrade on the primary outcome in patients with AF was persistent regardless of the NYHA subgroup, but the most prominent treatment effect could be observed in mildly symptomatic patients (NYHA class II OR 0.005; 95% CI 0.00–0.10; *P* < 0.001 and NYHA class III or IVa OR 0.07; 95% CI 0.02–0.29; *P* < 0.001). Moreover, this beneficial effect was also reflected in hard outcomes, the composite endpoint of all-cause mortality or HF hospitalization (HR 0.22; 95% CI 0.07–0.71; *P* = 0.011) and HF hospitalization alone (HR 0.25; 95% CI 0.07–0.81; *P* = 0.021).

### Outcomes in the cardiac resynchronization therapy with defibrillator arm by baseline rhythm

In the CRT-D upgrade arm, patients with atrial fibrillation experienced an almost three-fold higher hazard for the composite of all-cause mortality or HF hospitalization (adjusted HR 2.99; 95% CI 1.26–7.13; *P* = 0.013) (*Figure 2B*) as compared with those with SR and more than four-fold higher hazard for HF hospitalization alone (adjusted HR 4.52; 95% CI 1.54–13.33; *P* = 0.006). Patients with atrial fibrillation also encountered higher odds for worsening in NYHA functional class (NYHA change adjusted OR 2.80; 95% CI 1.52–5.13; *P* = 0.001).

Even though the rate of biventricular pacing was lower in the group of AF compared with SR [biventricular pacing rate at 12 months: SR 99% (IQR 97–100) vs. AF: 97% (IQR 92–99); *P* = 0.0029], almost 70% of the cohort reached a biventricular pacing rate more than 95%.

## Discussion

Patients in the BUDAPEST-CRT Upgrade trial showed a substantial treatment effect of CRT-D on the primary outcome regardless of the baseline rhythm. In addition, this pre-specified subgroup analysis of AF patients demonstrated:

- A clear benefit of CRT-D upgrade compared with ICD.
- Higher risk of HF hospitalization as compared with patients with SR.
- Atrial fibrillation or flutter patients in the CRT-D arm experienced improvements in echocardiographic parameters, NT-proBNP levels, and HF symptoms.

Since there have been no large randomized controlled trials directly comparing the effect of CRT in patients with AF vs. SR, the current guidelines refer a class IIa indication with level of evidence C for implanting a CRT in HFrEF patients with NYHA III–IV and wide QRS to improve symptoms and reduce morbidity and mortality.^5^ While these indications are derived from subgroup analyses of RCTs or observational studies,^15–17^ which mainly failed to show any benefits, the outcome of patients eligible for CRT implantation with AF remained inconclusive.^6,18^

In the subgroup analysis of the RAFT trial with permanent AF patients,^17^ those in the CRT-D group did not show a risk reduction on the primary endpoint of death or HF hospitalization as compared with ICD alone. The reasons behind these differences might be sought in low (<95%) biventricular pacing rate as a consequence of AF. In contrast, in the BUDAPEST-CRT trial, biventricular pacing burden >95% was achieved in 75% of AF patients, as in most patients, severely impaired atrioventricular conduction could be observed with an 85% median of RV pacing burden prior to enrolment. Our trial did not focus on the role of rate and rhythm strategies in AF patients. Atrial fibrillation or flutter was considered permanent with slow ventricular response in most patients, and this was also the original indication for pacing prior to enrolment into the trial. Despite the treatment effect of CRT-D upgrade, patients with AF still had a higher risk of having HF hospitalization as compared with those in SR. Therefore, maintaining SR may be crucial, but these findings still leave the question of rhythm control strategy in advanced HF open for further research.

The underlying causes behind the different treatment effect and outcome data in AF patients in previous trials can also be associated with the comorbidity burden. In a recent patient-level meta-analysis of four RCTs, in patients with CRT and a history of AF, CRT was not associated with improved outcomes.^18^ Thus, AF might have critically mitigated the efficacy of the device. These patients were older and had a higher burden of other comorbidities and showed an overall worse outcome.^18^ At the same time, in the BUDAPEST-CRT Upgrade trial including a very-advanced stage HF cohort with high age and a similarly high comorbidity burden,^13^ our results proved for the first time the substantial treatment effect of CRT in AF patients not only on hard outcomes (all-cause mortality and HF hospitalization) but also on echocardiographic improvement, HF symptoms, and NT-proBNP change. Therefore, we believe this contributes to the closure of an important evidence gap.

## Conclusions

Heart failure with reduced ejection fraction patients with AF and with high RV pacing burden showed remarkable risk reduction in HF hospitalizations or deaths, as well as an improvement in echocardiographic and functional outcomes after CRT-D vs. ICD upgrade. Nevertheless, patients with AF as compared with those in SR continued to have a higher risk for HF hospitalizations.

### Limitations

Some limitations of this sub-study should be noted. The specific inclusion and exclusion criteria may influence the results, and the number of subjects in different subgroups is limited. Echocardiographic response could not be evaluated when data were missing at any time point, but these rates were comparable in both AF and SR subgroups. The underlying causes of decreased biventricular pacing were not determined whether it was AF or any other factor (e.g. ventricular extrasystole). The treating strategy of the arrhythmias before or during the course of the trial was based on the physician’s discretion as per the current guidelines.^19–23^

## Supplementary Material

euae179_Supplementary_Data

## Data Availability

The data underlying this article will be shared on reasonable request to the corresponding author.
