# Supplementary material for: Benefits of upgrading right ventricular to biventricular pacing in heart failure patients with atrial fibrillation
Source: Europace. 2024 Jul 9;26(7):euae179. doi: 10.1093/europace/euae179 (PMC11264293; doi:10.1093/europace/euae179)
Supplement: euae179_Supplementary_Data [file euae179_supplementary_data.docx]

**Supplementary Material**

Supplementary Figure 1. Kaplan Meier Curves of the three groups by the presence and history of atrial fibrillation (AF) in the CRT-D arm
